# The intersection between pre-existing disability and cancer-survivorship: A narrative review

**DOI:** 10.34172/hpp.025.43001

**Published:** 2025-05-06

**Authors:** Winston Kennedy, Jafra D Thomas, Gullaiim Almatkyzy, Regina F. Hockert

**Affiliations:** ^1^Department of Physical Therapy Movement and Rehabilitation Sciences, Bouve College of Health Sciences, Northeastern University, Boston, MA, USA; ^2^Department of Kinesiology and Public Health, California Polytechnic State University, San Luis Obispo, CA, USA; ^3^Department of Oral Health Sciences, University of Washington, WA, USA; ^4^California Polytechnic State University, San Luis Obispo, CA, USA

**Keywords:** Health promotion, Quality of life, Rehabilitation

## Abstract

**Background:**

Previous research shows people with pre-existing disabilities face unique societal barriers to health services. The purpose of this review is to promote a better understanding of ways pre-existing disability affects access to cancer-related care through a critical commentary of the research literature.

**Methods:**

Systematic search procedures were used to locate articles for inclusion in a narrative review of the research literature. A priori search terms were used to systematically search for eligible articles, using four online databases. These authors thematically analyzed trends in the research findings using a descriptive-interpretive paradigm.

**Results:**

Thirteen articles were included in the final narrative review. Four themes were identified: (1) low screening rates, (2) barriers to cancer screening, (3) pre-existing disability moderates cancer incidence, treatment, and survival, and (4) recommendations for supporting improved HRQoL in cancer survivors with pre-existing disability.

**Conclusion:**

Our findings illustrate a need for interdisciplinary research focused on ways pre-existing disability intersects with cancer-related healthcare.

## Introduction

 People with disabilities fare far worse on various health indicators when compared to people without disabilities.^1^ They have higher rates of obesity, smoking, diabetes, cardiovascular diseases, and engage in physical activity (PA) less when compared to their non-disabled counterparts.^1^ These factors are also associated with the diagnosis of cancer.^2^ According to the 2016 Behavioral Risk Factor Surveillance System (BRFSS) survey, 25.7% of noninstitutionalized U.S. adults (representing an estimated 61.4 million persons) reported having a disability.^3^ Mobility was the most prevalent disability type (13.7%), followed by cognition (10.8%), independent living (6.8%), hearing (5.9%), vision (4.6%), and self-care (3.7%).^3^ Until recently, there has been little investigation on the intersection of cancer and pre-existing disability as it relates to health.^2,4-6^ Also, there has been very little research on pre-existing disability and cancer survivorship outcomes including the health-related quality of life (HRQoL) of persons with cancer and other disabilities.^7^

 A person is considered a ‘cancer survivor’ from the time of cancer diagnosis through the end of their life.^8^ There are around 17 million cancer survivors in the United States, and it is expected to grow to more than 22 million by 2030 due to public health efforts such as early screening and detection and improved cancer treatments.^9,10^ In men, prostate, colorectal, and melanoma are the most prevalent cancers, whereas females are commonly diagnosed with breast, uterine corpus, and colorectal cancers.^9^ Despite the advancements in cancer treatment and outcomes, cancer survivors are susceptible to secondary cancers, cancer recurrence, and may experience late effects of cancer treatment.^11^ Some of the short- and long-term health effects of cancer and its treatment include impaired physical functioning for breast cancer survivors, neuropathy and bowel dysfunction for colorectal cancer survivors, physical impairment, and sexual problems for prostate cancer survivors.^9^ Moreover, cancer survivors report worsened general health and experience diminished emotional well-being than the general population.^12^ These physical and mental sequelae may have negative effects on cancer survivors’ HRQoL, including physical, functional, emotional, and social well-being.^13^

 PA has long been reported as a method to reduce risk and mortality with cancer.^14^ With the consistent increase in cancer survivors, the American College of Sports Medicine (ACSM) convened a Roundtable to develop a set of exercise guidelines for cancer survivors in 2010. Through their interprofessional collaboration, the ACSM Roundtable concluded that PA was safe for cancer survivors and that sedentary behaviors should be limited.^15^ In 2018 a Roundtable of international and interprofessional scholars convened to advance the ACSM Roundtable guidelines to be more specific to cancer type, treatments, and outcomes.^16^ Similar to the ACSM Roundtable, it was concluded that when dosed appropriately PA could improve HRQoL in cancer survivors such as decreased anxiety, depression, and fatigue, and improved overall physical functioning.^16^ Despite the documented benefits and reported guidelines to support engagement, PA engagement for cancer survivors is poor.^17^ The lack of PA engagement in cancer and the associated health risks of cancer survivors may be compounded by the health effects of having a pre-existing disability.

 Pre-existing disabilities have long-term effects for cancer survivors’ HRQoL, especially for physical well-being.^7^ For instance, individuals with multiple sclerosis have increased fatigue after cancer treatment compared to their counterparts without multiple sclerosis ^7^ which could further diminish their physical and functional well-being. When women with pre-existing mobility disability receive radiation therapy and chemotherapy for breast cancer they might later show side effects such as lymphedema, which further hinder wheelchair use.^7^ Furthermore, a recent study found that individuals with pre-existing disabilities experience acute anxiety, depression, and a sense of loss of control over their health conditions when they are diagnosed with cancer,^4^ which might worsen their emotional well-being. Although cancer survivorship studies have focused on barriers and facilitators of HRQoL of survivors, to our knowledge, there is limited research on the intersection of pre-existing disability and cancer survivorship that support HRQoL of cancer survivors. Therefore, the purpose of this narrative review is to promote a better understanding of ways pre-existing disability affect the access to cancer-related health services in this population, and to identify recommendations to improve care.

## Methods

**Figure 1:**
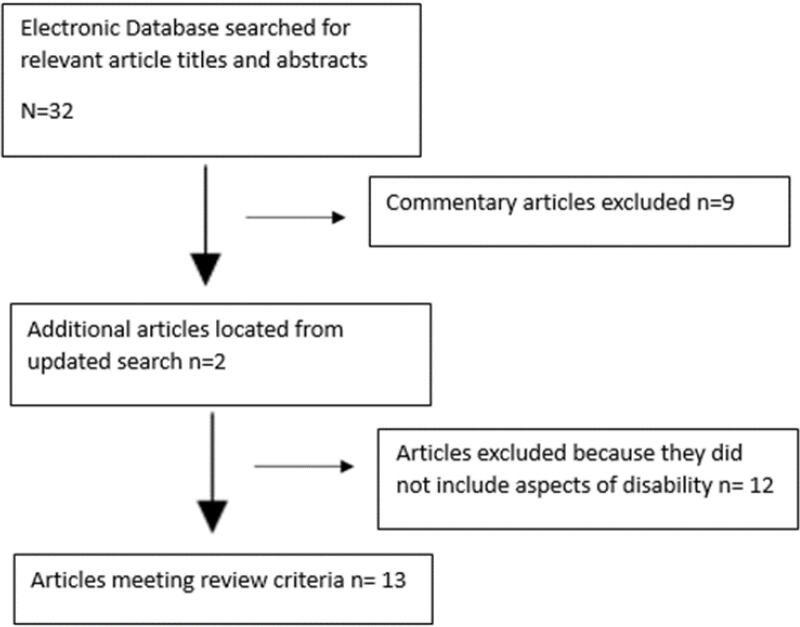
Flow diagram of the identification and selection of studies included in review

 Given this study did not involve human subjects, review and pre-approval of its protocol by the authors’ institutional review board was not required. In identifying sources for the narrative review four electronic databases were used: “PubMed”, “Medline”, “Google Scholar”, and “PsychInfo”. The first and third author searched for articles, and then came to a consensus on which articles were included. The inclusion criteria for peer-reviewed articles to be included were they had to be written in English and must have focused on pre-existing disability and cancer-related health and healthcare in the United States. Randomized control studies, cross-sectional/observational studies, guidelines and reviews were all considered for inclusion. The exclusion criteria for articles were if articles focused on cancer related health and healthcare for people who became disabled after cancer diagnosis. Search terms included “cancer survivors”, “cancer treatment”, “cancer screening” AND “disability”, “pre-existing disability”. Search terms were intentionally kept broad to be able to conduct a wider search. The snowball method was used to include additional articles (i.e., checking the reference list for other sources to potentially include in the review). The second author served as a critical friend to review the inclusion of articles, in order to independently verify the research report and provide an outside perspective to reduce researcher bias.^18^ See Figure 1 for the identification and selection of studies included in review.

## Results

 As there is shown in Table 1, thirteen articles were included in the narrative review (*M* publication date = 2012.67, *SD* = 5.66). Four themes were identified when data was extracted and analyzed for trends. The four themes conceptualized from the qualitative trends were: people with pre-existing disability have (1) low screening rates (2) and barriers to cancer screening, (3) pre-existing disability moderates cancer incidence, treatment, and survival, and (4) Recommendations for supporting improved HRQoL in cancer survivors with pre-existing disability.

**Table 1:** Results of Search

**Theme**	**Article title **	**Data**	**Reference**
Lower screening rates	Prevalence of cancer screening among adults with disabilities, United States	Secondary data analysis 2013 National Health Interview Survey data Cross-sectional study	^19^
Barriers to cancer screening	Obstacles to preventive care for individuals with disability: Implications for nurse practitioners	Literature Review	^20^
	Barriers to cancer screening for people with disabilities: A literature review	Literature review	^21^
	Health and wellness: People with disabilities discuss barriers and facilitators to well being	Qualitative study	^22^
	Barriers to, and facilitators of, access to cancer services and experiences of cancer care for adults with a physical disability: A mixed methods systematic review	Systematic review	^23^
Impact of pre-existing disabilities on cancer incidence, treatment, and survival	Cross-sectional analysis of the associations between four common cancers and disability	Cross-sectional study	^2^
	Predictors of quality of life for long-term cancer survivors with pre-existing disabling conditions	Cross-sectional study	^7^
	Early-stage breast cancer treatments for younger Medicare beneficiaries with different disabilities	Retrospective cohort study	^24^
	Disparities in breast cancer treatment and survival for women with disabilities	Retrospective cohort study	^25^
Recommendations for supporting increased HRQoL	Exercise guidelines for cancer survivors: consensus statement from international multidisciplinary roundtable	Scoping Review	^16^
	A double whammy: health promotion among cancer survivors with preexisting functional limitations	Qualitative Study	^6^
	Review of systematic reviews of non-pharmacological interventions to improve quality of life in cancer survivors	Scoping Review	^26^
	Lost in transition: an American Society of Clinical Oncology and Institute of Medicine symposium	Executive Summary	^27^
	Health and wellness: People with disabilities discuss barriers and facilitators to well being	Qualitative study	^22^

###  Cancer screening rates among people with pre-existing disability 

 Through our review we found one article that investigated the screening rates for cervical, breast, and colorectal cancers for people with and without disabilities.^19^ Steel and colleagues conducted a secondary data analysis using the 2013 National Health Interview Survey data and found that women with cognitive and vision disabilities are less likely to get screened for breast cancer than women without a disability.^19^ Furthermore, women with mobility disabilities have a lower rate of reporting having had a Papanicolaou (Pap) test for cervical cancer, when compared to women with other types of disabilities and women without disabilities. Similarly, people with vision and cognitive disabilities report lower rates of colorectal cancer screening than people with hearing and mobility disability, and people without disabilities. The findings of this study suggest that people with disabilities are less likely to get cervical, breast, and colorectal cancer screening than people without disabilities.

###  Barriers to cancer screening among people with pre-existing disabilities

 There were four articles that discussed barriers to cancer screening. The earliest study was a qualitive study published in 2003 and the most recent article was a systematic review published in 2020.^20-23^ Two of the four studies focused specifically on cancer screening but more broadly focused preventive health, however, since cancer screening is a form of preventive health care, these were included. There was consistency across studies on barriers for preventative care and specifically cancer screening. Common themes included accessibility, finances, healthcare provider knowledge and attitudes.^20-23^ Within accessibility, specific barriers include physical access to facilities (e.g., ramped access), transportation, equipment (e.g., specialized hospital treatment tables), and overall poor health systems (e.g., scheduling appointments, poor care coordination with providers, and general additional assistance). Within finances, specific barriers include lack of money and health insurance coverage to address health needs. Lastly, within healthcare provider knowledge and attitudes, specific barriers include lack of knowledge as it pertains to health of people with disabilities, biased health information due to race, gender, and/or type of disability.

###  Impact of pre-existing disabilities on cancer incidence, treatment, and survival

 Four articles were included that discussed the impact of pre-existing disabilities on cancer incidence, treatment, and survival. Two articles focused specifically on breast cancer.^24,25^ In both studies women with disabilities (such as women with mental disorders and neurological conditions) were less likely to receive breast conserving surgery and radiation when compared to women without disabilities. Women with mental disorders also had a higher mortality rate than women without disabilities. Another article focused on investigating associations between different types of cancers and disability.^2^ The study used a national sample of non-institutionalized adults who responded to the National Health Survey. It was suggested that people with pre-existing disabilities have a higher incidence of cancer than those without disability. It was also found that people with movement difficulties were 1.5 times more likely to get colorectal cancer than those without disability. Similarly, persons with complex activity limitations were 1.9 times more likely to get colorectal cancer than those without one. Other behavioral and socioeconomic factors that are common in people with disabilities that might affect cancer incidence include obesity, tobacco smoking, and poverty. The authors of the study indicated that diagnostic overshadowing, a practice where new symptoms of cancer are misinterpreted as patients’ underlying chronic disability, might have played a role in late cancer diagnosis among people with physical, developmental, and intellectual disability, although this requires further investigation. Lastly, one study investigated predictors of quality of life for cancer survivors with pre-existing disability.^7^ They surveyed 145 participants and found that cancer survivors with pre-existing disabilities reported worse physical well-being when compared to cancer survivors without pre-existing disabilities. It was also suggested that increased engagement in health-promoting behaviors and positive psychosocial factors (e.g., high self-efficacy) increases the likelihood of increased positive physical, social, emotional, and functional components of HRQoL.

###  Recommendations for supporting improved HRQoL in cancer survivors with pre-existing disability

 Five articles included recommendations for supporting improved HRQoL for people with and without pre-existing disability. While some guidelines were not specifically for cancer survivors with pre-existing disability, the recommendations were relevant for the population. One article investigated the barriers to and facilitators of health and well-being for people with disabilities.^22^ While, the article did not pertain specifically to cancer survivors, it did discuss recommendations to facilitate better healthcare access and experiences (e.g., increasing health professional knowledge on health, wellness and disability), which also benefits the increased HRQoL for cancer survivors with pre-existing disabilities. There was one article that explored the experiences of cancer-survivors who had a pre-existing disability using qualitative research methods^6^; authors suggested that health promotion efforts need to be tailored to support cancer survivors with pre-existing disabilities because of their unique needs. One article was an executive summary that provided evidenced based recommendations to improve the HRQoL of cancer survivors.^28^ One article was a systematic review that addressed interventions to increase HRQoL, discussed the impact of psychological/behavioral interventions, and suggested that cognitive behavioral therapy, can effectively improve HRQoL in both the short and long term.^26^ The last and most recent article provided specific details with their recommendations of PA, including exercised dosage and types of activity.^16^

## Discussion

 The purpose of this narrative review was to promote a better understanding of ways pre-existing disability impacts the access to and quality of cancer-related health services in cancer survivors, while also investigating recommendations to improve care. This narrative review also begins to establish a broad overview of how pre-existing disability intersects with cancer survivorship and HRQoL. Four themes were identified in the synthesis of articles included in the review: low cancer screening rates for people with pre-existing disabilities, barriers to cancer screenings among people with pre-existing disability, the impact of pre-existing disabilities on HRQoL of cancer survivors, and recommendations to improve their HRQoL for cancer survivors with pre-existing disability.

 There is a growing number of cancer survivors in the US population, including those with pre-existing disability,^9^ however, there are some distinct differences in access to cancer related screenings and services when comparing cancer survivors with and without pre-existing disabilities.^2,7,19^ Individuals with pre-existing disabilities have lower rates of cancer screening than those without disabilities, which makes it hard to treat cancer when found later in life. Steele et al^19^ examined the prevalence of cancer screening tests by disability type and found that women with cognitive and visual disabilities are less likely to get screened for breast cancer than women without a disability. Furthermore, women with mobility disabilities have a lower rate of reporting having a screening for cervical cancer when compared to women with other types of disabilities and women without disabilities. Similarly, people with visual and cognitive disabilities report lower rates of screening for colorectal cancer than people with hearing and mobility disability, and people without disabilities. This was the only comprehensive study located by our systematic search that investigated cervical, breast, and colorectal cancer screenings procedures for people with various disabilities. One study investigated the use of colorectal cancer screening among people with mobility disability, and the study results suggested that people with mobility disability have reduced colorectal cancer screenings when compared to other groups.^29^ An updated investigation into the frequency of cancer screenings should be investigated.

 Low screening rates for cancer survivors for people with pre-existing disabilities led to the identification of barriers to getting cancer screenings. Some of the existing barriers for people with pre-existing disabilities include challenges with getting cancer screening appointments, wait time in the clinic, and lack of transportation, which are social determinants of health contributing to inequities in cancer care for people with pre-existing disabilities.^21,23^ It has also been suggested that that healthcare providers often feel discomfort and do not have the knowledge and communication training when working with people with disabilities generally,^20,22^ and specifically on the need for cancer screening for people with disabilities.^21,23^ Moreover, patients with pre-existing disabilities have indicated their frustration about the lack of information about why and how to get screened for cancer including the logistics of the screening process.^21^ Another major barrier is the attitudes of health professionals towards people with disabilities. For instance, nurses tend to focus their attention on individuals’ disabilities, thereby forgetting to assign cancer screening services and provide recommendations about preventive health practices.^20^ Other barriers to cancer screening include physically inaccessible healthcare facilities, lack of social and interpersonal support, and financial constraints.^23^ While there has been some work to increase the rates of cancer screenings for people with disabilities,^30,31^ the implementation and impact of such programs have not been investigated thoroughly.

 People with pre-existing disabilities have a higher incidence of cancer than those without disability,^2,25^ highlighting the major impact of pre-existing disability on cancer in US society as a result of institutional and interpersonal discrimination. A recent study that used a national sample of non-institutionalized adults found that people with movement difficulties were 1.5 times more likely to get colorectal cancer than those without disability.^2^ Regarding breast cancer treatment, women with pre-existing disabilities were less likely to receive standard therapy (such as radiotherapy and axillary lymph node dissection) after breast-conserving surgery than women without disabilities.^25^ Furthermore, women with mental and intellectual disabilities, and neurological conditions who were diagnosed with early-stage cancer were less likely to receive breast-conserving surgery and radiotherapy when compared to women without disabilities.^24^ The disparities in cancer incidence, treatment and survival highlight the growing need for specific but evidence-based care for people with pre-existing disabilities regarding cancer screening, treatment and survivorship care. In our investigation we found recommendations that may help with the cancer disparities experienced by people with pre-existing disabilities.

 Existing recommendations to improve HRQoL for cancer survivors with pre-existing disabilities varied. PA recommendations for cancer survivors did not specifically focus on people with pre-existing disabilities, however some of the guidelines could apply (e.g., promotion of activity, fatigue management).^16^ However, because there is not an emphasis on pre-existing disability, recommendations may not be applicable for the population, (i.e., lack of knowledge by health professionals on PA for people with disabilities).^32,33^ Specific PA guidelines for cancer survivors with pre-existing disabilities should be developed. Further, there is a need to improve shared post-treatment care and coordination among healthcare professionals. This could be accomplished through disability-related communication training for health professionals that promote working with cancer patients with pre-existing disabilities.^6,27^ For instance, a nurse navigation services program that coordinates care for recently diagnosed cancer patients has the potential to improve patient-provider communication and increase survivors’ HRQoL and satisfaction with care.^6,34^

## Conclusion

 Outside of PA promotion, broader health promotion programs that include tailored informational (about post-treatment life) and instrumental (e.g., accessible environment for follow-up care, appropriate PA and nutritional information to meet special needs) support from healthcare professionals and social support from family and friends would help support HRQoL.^35^ Cancer survivors with pre-existing disabilities may also struggle with financial hardship due to cancer treatment on top of expenses that may incur with managing a pre-existing disability^6^; therefore, disability-inclusive health-promotion programs at safety-net health centers should be implemented as well. Lastly, most cancer survivorship research usually do not include questions that evaluate survivors’ pre-existing disabilities^2,6^; therefore, future studies should include validated measurement to assess prior disability-related conditions.

## Limitations

 This narrative review study is not without its limitations, which should guide the interpretation of its results and give direction to future research. First, only one study included in this review examined screening rates for colorectal cancer using the 2013 dataset of the National Health Interview Survey.^19^ This limited this review’s ability to promote awareness of disparities in cancer screening rates for different forms of cancer for individuals with pre-existing disabilities. Second, future review studies should explore if published studies exist analyzing more recent datasets of the National Health Interview Survey.^36^ Additionally, future review studies should seek published studies that analyze other national datasets that include questions specific to cancer screening rates and disability status, such as the Behavioral Risk Surveillance System Survey.^37^ It is important to also note that the present review study was limited to studies that sampled adults residing within the United States.

 Third, studies included in this review employed a limited utilization of intersectionality theory.^38^ Future review studies should deliberately seek investigations that examine disparities and experiences inclusive of other intersectional identities beyond cisgender gender identity and disability type. Other intersecting identities with disability include social economic status, race/ethnicity, sexual orientation, and non-binary gender identities. Finally, as a narrative review, the aims of this study was to promote awareness of how having a pre-existing disability could relate to cancer screening rates and healthcare quality focused on cancer prevention and treatment. Thus, a final limitation to note is that this study did not systematically critically appraise the publications included in this review, nor did it synthesize study findings beyond a descriptive-thematic analysis. Future review studies should analyze their included studies using techniques for critical appraisal and research synthesis.^39^

## Competing Interests

 None.

## Ethical Approval

 Not applicable.
